# Supplementary material for: Perceptual saccadic suppression starts in the retina
Source: Nat Commun. 2020 Apr 24;11:1977. doi: 10.1038/s41467-020-15890-w (PMC7181657; doi:10.1038/s41467-020-15890-w)
Supplement: Supplementary file 3 — Reporting Summary [file 41467_2020_15890_MOESM3_ESM.pdf]

nature research

Corresponding author(s): Ziad M. Hafed
Thomas A. Münch

Last updated by author(s): Mar 19, 2020

Reporting Summary

Nature Research wishes to improve the reproducibility of the work that we publish. This form provides structure for consistency and transparency in reporting. For further information on Nature Research policies, see Authors & Referees and the Editorial Policy Checklist.

Statistics

For all statistical analyses, confirm that the following items are present in the figure legend, table legend, main text, or Methods section.

n/a	Confirmed	
☐	☒	The exact sample size (*n*) for each experimental group/condition, given as a discrete number and unit of measurement
☐	☒	A statement on whether measurements were taken from distinct samples or whether the same sample was measured repeatedly
☐	☒	The statistical test(s) used AND whether they are one- or two-sided<br>*Only common tests should be described solely by name; describe more complex techniques in the Methods section.*
☒	☐	A description of all covariates tested
☐	☒	A description of any assumptions or corrections, such as tests of normality and adjustment for multiple comparisons
☐	☒	A full description of the statistical parameters including central tendency (e.g. means) or other basic estimates (e.g. regression coefficient) AND variation (e.g. standard deviation) or associated estimates of uncertainty (e.g. confidence intervals)
☐	☒	For null hypothesis testing, the test statistic (e.g. *F*, *t*, *r*) with confidence intervals, effect sizes, degrees of freedom and *P* value noted<br>*Give P values as exact values whenever suitable.*
☒	☐	For Bayesian analysis, information on the choice of priors and Markov chain Monte Carlo settings
☒	☐	For hierarchical and complex designs, identification of the appropriate level for tests and full reporting of outcomes
☒	☐	Estimates of effect sizes (e.g. Cohen's *d*, Pearson's *r*), indicating how they were calculated

Our web collection on statistics for biologists contains articles on many of the points above.

Software and code

Policy information about availability of computer code

Data collection	Retina ephys recordings: MC Rack version 4.6.2, MEA 1k Scope; Human psychophysics: MATLAB
Data analysis	MATLAB

For manuscripts utilizing custom algorithms or software that are central to the research but not yet described in published literature, software must be made available to editors/reviewers. We strongly encourage code deposition in a community repository (e.g. GitHub). See the Nature Research guidelines for submitting code & software for further information.

Data

Policy information about availability of data

All manuscripts must include a data availability statement. This statement should provide the following information, where applicable:

- Accession codes, unique identifiers, or web links for publicly available datasets
- A list of figures that have associated raw data
- A description of any restrictions on data availability

All data are stored and archived on secure institute computers, and they are available upon reasonable request.

Field-specific reporting

Please select the one below that is the best fit for your research. If you are not sure, read the appropriate sections before making your selection.

☒ Life sciences ☐ Behavioural & social sciences ☐ Ecological, evolutionary & environmental sciences

For a reference copy of the document with all sections, see nature.com/documents/nr-reporting-summary-flat.pdf

Life sciences study design

All studies must disclose on these points even when the disclosure is negative.

Sample size	No sample size calculations were performed. For each ganglion cell recorded, responses were averaged in most cases 39 independent observations, for every condition that we tested. In some cases there were less observations. In any case, an effect in the responses was reported as significant only if the power of the statistical test was greater than 80% given the exact number of observations. This was done for all the 1,064 ganglion cells which were used in different analysis of this paper. For analysis reporting average across cells, we have N numbers >300 cells for most crucial conditions. These sample sizes are sufficient because under baseline conditions, their normalized responses are normally distributed (Supplementary Fig. 5b, c, last column). Furthermore, in our figures, variability in population is shown.<br><br>In most human psychophysics experiments, we used 6-8 human subjects. We report the variability across subjects and also show data for each individual subject. Moreover, as per reviewer suggestions, we performed extensive psychometric experiments (which gives a more strict measure) and analyses to confirm our findings. These are generally accepted N numbers given the low variability observed and exhaustive experiments.
Data exclusions	No data excluded
Replication	In case of retina electrophysiology, to acquire data, the same experiments were repeated several times with different retinae, different recording devices and different stimulating devices. In total we performed 20 independent experiments from mice retinae and 12 independent experiments from pig retinae. Each experiment, contained the basic paradigm described in this paper and therefore we could pool the cells recorded across these experiments. Additional 4 experiments with a different paradigm were later added during the review process. Cells were also pooled across these experiments. We did not find any differences in experiments using different recording or stimulating devices.<br><br>In the case of human psychophysics, each subject was measured for multiple sessions and several subjects were measured. The same subjects performed multiple versions of the experiments to demonstrate robustness of the differential effects across versions. The number of sessions varied across experiments and subjects. In general, 2-10 sessions (45 minutes per session) of each experiment was repeated on 6-8 subjects.
Randomization	Trials (with different conditions) presented to retina in electrophysiology experiment were randomized to avoid adaptation to any condition. Trials presented to human subjects in psychophysics were randomized to avoid subjects remembering any order. Moreover, multiple background images were used for similar reasons.
Blinding	Blinding was not required as conditions need not be tested on different groups.

Reporting for specific materials, systems and methods

We require information from authors about some types of materials, experimental systems and methods used in many studies. Here, indicate whether each material, system or method listed is relevant to your study. If you are not sure if a list item applies to your research, read the appropriate section before selecting a response.

Materials & experimental systems

n/a	Involved in the study	
☒	☐	Antibodies
☒	☐	Eukaryotic cell lines
☒	☐	Palaeontology
☐	☒	Animals and other organisms
☐	☒	Human research participants
☒	☐	Clinical data

Methods

n/a	Involved in the study	
☒	☐	ChIP-seq
☒	☐	Flow cytometry
☒	☐	MRI-based neuroimaging

Animals and other organisms

Policy information about studies involving animals; ARRIVE guidelines recommended for reporting animal research

Laboratory animals	We used retinae extracted from PV-Cre x Thy-S-Y male and female mice 3-12 months old. Mice were housed on a 12/12 h light/dark cycle in ambient temperature, ranging between 20-22 °C, and humidity levels of approximately 40%. Mice were dark adapted for 4-16 h before experiments.<br><br>We also used pig retinae obtained from domestic female pigs after they had been sacrificed during independent studies at the Department of Experimental Surgery in our Medical Faculty. We have little information on the exact age of the pigs but we only used those retinae for experiments which seemed healthy and showed light responses. In total, we did 12 recordings from retinae of 6 pigs and results were consistent across retinae of different pigs.
Wild animals	No wild animals were used in this study. We used retinae from 6 domestic pigs which were bred for experiments.
Field-collected samples	No field collected samples were used in this study.

Ethics oversight	Animal use was in accordance with German and European regulations, and animal experiments were approved by the Regierungspräsidium Tübingen.

Note that full information on the approval of the study protocol must also be provided in the manuscript.

Human research participants

Policy information about studies involving human research participants

Population characteristics	We collected data from healthy adult subjects, aged 21-29 years. In each experiment, we reported the number of female and male subjects in Methods.
Recruitment	Human subjects provided written, informed consent, and they were paid 8-15 Euros per session of 45-90 minutes each. The subjects were recruited through university mailing lists and social media related to psychophysical experiments at the university. The subjects were therefore primarily students. We did not exclude any specific populations otherwise. Given that students at an international university are representative of normal/healthy populations of their age group, the fact that most subjects were likely to be students is unlikely to affect our interpretations. In addition, the phenomena that we studied have been reported robustly previously in other studies, both for similar age groups as well as other older ones.
Ethics oversight	Human experiments were approved by ethics committees at the Medical Faculty of Tübingen University, and they were in accordance with the Declaration of Helsinki.

Note that full information on the approval of the study protocol must also be provided in the manuscript.
